# Assessing cultural safety in general practice consultations for Indigenous patients: protocol for a mixed methods sequential embedded design study

**DOI:** 10.1186/s12909-023-04249-6

**Published:** 2023-05-02

**Authors:** Kay Brumpton, Raelene Ward, Rebecca Evans, Henry Neill, Hannah Woodall, Lawrie McArthur, Tarun Sen Gupta

**Affiliations:** 1 Rural Medical Education Australia, Toowoomba, Australia; 2 University of Southern Queensland, Toowoomba, Australia; 3 College of Medicine and Dentistry, James Cook University, Townsville, Australia

**Keywords:** Cultural safety, General practice, Registrars, Indigenous, Assessment

## Abstract

**Background:**

Assessment of cultural safety in general practice consultations for Indigenous patients is a complex notion. Design and development of any assessment tool needs to be cognizant that cultural safety is determined by Indigenous peoples and incorporates defined components of cultural safety and current educational theory. Consideration of how social, historical, and political determinants of health and well-being impact upon the cultural safety of a consultation is also important. Given this complexity, we assume that no single method of assessment will be adequate to determine if general practice (GP) registrars are demonstrating or delivering culturally safe care. As such, we propose that development and assessment of cultural safety can be conceptualised using a model that considers these variables. From this, we aim to develop a tool to assess whether GP registrars are conducting a culturally safe consultation, where cultural safety is determined by Aboriginal and Torres Strait Islander peoples.

**Methods:**

This protocol will be situated in a pragmatic philosophical position to explore cultural safety primarily from the Australian Aboriginal and Torres Strait Islander patients’ perspective with triangulation and validation of findings with the GP and GP registrar perspective, the Aboriginal and Torres Strait Islander community, and the medical education community. The study will integrate both quantitative and qualitative data through three sequential phases. Data collection will be through survey, semi-structured interviews, an adapted nominal group technique, and a Delphi questionnaire. We aim to recruit approximately 40 patient and 20 GP participants for interviews, conduct one to five nominal groups (seven to 35 participants) and recruit fifteen participants for the Delphi process. Data will be analysed through a content analysis approach to identify components of an assessment of cultural safety for GP registrars.

**Discussion:**

This study will be one of the first to explore how cultural safety, as determined by Indigenous peoples, can be assessed in general practice consultations. This protocol is shared to stimulate awareness and discussion around this significant issue and prompt other studies in this area.

**Supplementary Information:**

The online version contains supplementary material available at 10.1186/s12909-023-04249-6.

## Background

Much has been written about the concept of cultural safety and its importance in the improvement of health care delivery for racial minority groups, particularly for Indigenous peoples affected by colonisation [1, 2]. Ramsden, a Māori nurse, who first proposed the concept of cultural safety in healthcare, articulated a three-step progression in the development of cultural safety from cultural awareness to sensitivity, and safety [3]. More recently, development of cultural safety has been described as a continuous circular model from awareness (cognizant of differences) to sensitivity (understanding and respecting these differences), competence (responding to own bias and developing skills), and safety [4]. Others, including Paul et al. [5], argue however that any discussion on cultural safety that focuses on differences and disparity between cultures, rather than reflection on practice, should be challenged.

Indeed, cultural safety is a complex notion and lack of a consistent definition has somewhat stymied the progression of evidence in this space [6]. Currently, whilst the concept of cultural safety or similar [7, 8] is embedded deeply in health and health education policy and frameworks [9–13] there is minimal evidence for the effectiveness of cultural safety training in improving patient health outcomes [14–18]. The use of a consistent definition for cultural safety provides an opportunity to provide clarity around the terminology used and address the paucity of evidence related to cultural safety [14–18]. Australia has attempted to address this by releasing a consensus statement, agreed upon by the Australian Health Practitioner Regulation Agency (AHPRA), regarding Australia’s colonised Indigenous population of Aboriginal and Torres Strait Islander peoples [19]:

“*Cultural safety is determined by Aboriginal and Torres Strait Islander individuals, families and communities. Cuturally safe practise is ongoing critical reflection of health practitioner knowledge, skills, attitudes, practising behaviours and power differentials in delivering safe, accessible and responsive healthcare free of racism*” [19].

According to the Australian Bureau of Statistics, Australian Aboriginal and Torres Strait Islander people represent approximately 3.2 percent of the Australian population [20] and have a burden of disease 2.3 times that of non-Indigenous Australians [21]. Aboriginal and Torres Strait Islander peoples’ strength and resilience, as one of the oldest world cultures, is impacted by the colonisation of Australia. Colonisation is described by Aboriginal health academic McKivett as: “the colliding of two worlds and the meeting of different systems of knowledges and beliefs” [8] p596. Colonisation, along with social determinants of health, continue to affect the health and wellbeing of Aboriginal and Torres Strait Islander peoples today. As such, Aboriginal and Torres Strait Islander people, again as described by McKivett et al., “Are striving to maintain collective values, traditions and beliefs whilst also coping with high burdens of chronic disease, reduced life expectancies and the impacts of grief, loss and trauma" (p596).

In Australia, AHPRA worked with other significant stakeholders to develop a national Aboriginal and Torres Strait Islander Health and Cultural Safety Strategy (2020–2025) which casts a vision that patient safety, including both clinical and cultural safety, is the expected standard of care for Aboriginal and Torres Strait Islander peoples and that this standard of safety is defined by Aboriginal and Torres Strait Islander peoples [22]. The National Strategy also considers that developing a culturally safe general practice workforce is a key strategy in improving health outcomes for Aboriginal and Torres Strait Islander peoples [22]. Delivery of culturally safe health care can improve quality of health care [23] and should, by inference, improve disparities in the life expectancy and morbidity patterns experienced by Indigenous peoples [24, 25]. For most patients in the developed world, a General Practitioner (GP) is the first point of contact when accessing healthcare and is the most persistent relationship for a patient within the health system [26]. As such, a culturally safe GP can play a crucial role to closing the gap in health outcomes for Indigenous peoples [27]. We argue that development of cultural safety in GP registrars is a priority for general practice training [28]. Logically, the development of cultural safety should occur early in postgraduate training as reflected by the recent inclusion of the AHPRA definition of cultural safety into the Australian GP curricula by the Royal Australian College of General Practitioners (RACGP) and the Australian College of Rural and Remote Medicine (ACRRM) [29, 30]. The AHPRA consensus statement, along with recent educational developments (for example, revision of Australian Medical Council graduate outcomes [31], and assessment changes by the RACGP [28]), provides opportunity to reassess the health professional educational response to teaching and assessment of cultural safety.

Understanding how GPs and GP registrars define, develop, and perceive cultural safety could assist identification of areas where cultural safety is lacking or needs improvement. A 2016 integrative review, conducted prior to release of the AHPRA definition, demonstrated a significant gap in evidence on how cultural safety is developed by GPs when consulting with Aboriginal and Torres Strait Islander peoples [32]. The literature cited suggests a lack of understanding regarding cultural safety development in GP registrars, or how cultural safety training influences GP registrar behaviour and consulting practices. Furthermore, as assessment is considered to drive learning, consideration of how cultural safety can be assessed, in a formative and summative manner, is required. These assessments require a clear definition and description of culturally safe practice, which must be determined by Aboriginal and Torres Islander people and communities themselves. We are not aware of any assessment tools of consultation skills based on community-derived definitions.

**Fig. 1:**
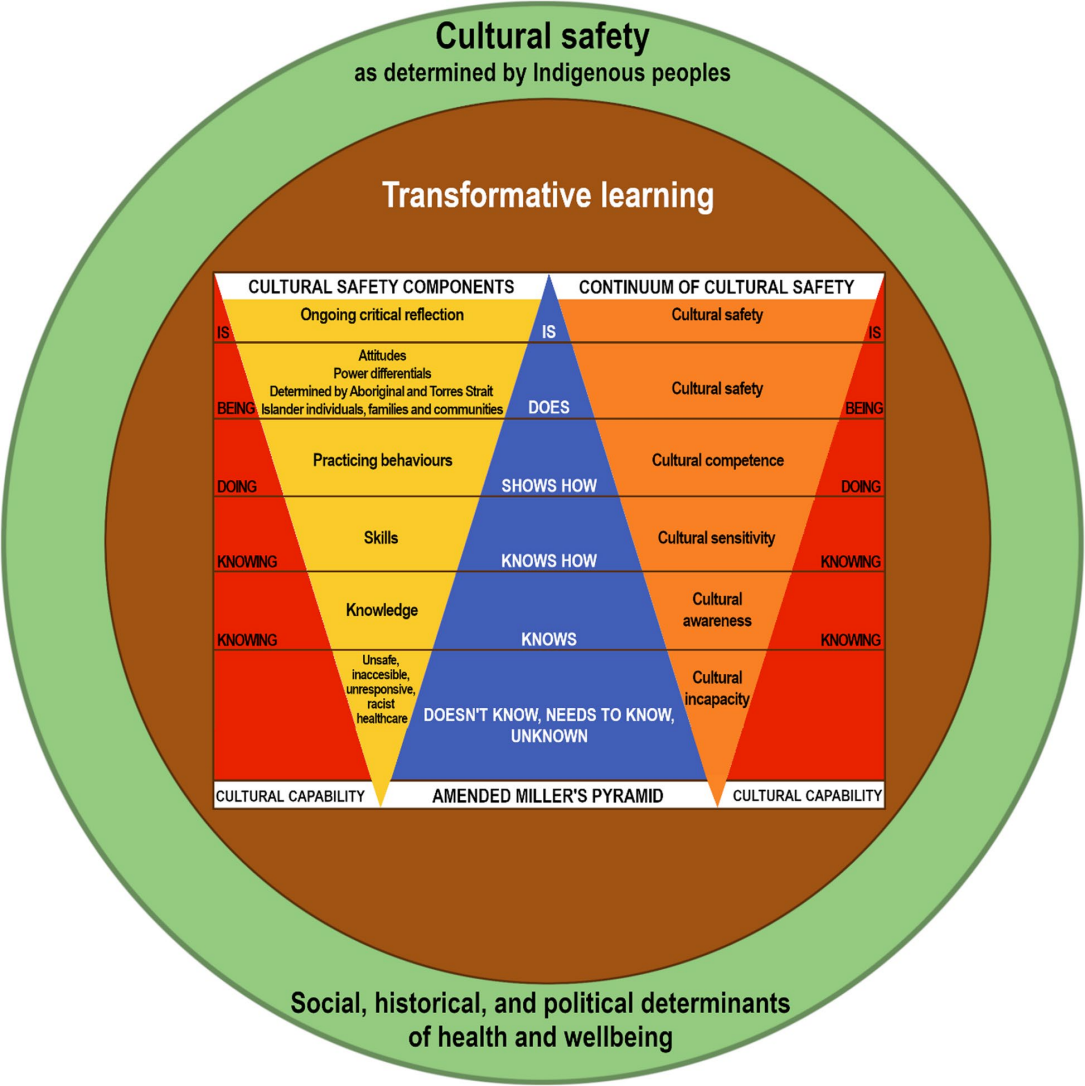
Proposed model of cultural safety in general practice

Given the complexity of cultural safety, we assume that no single method of assessment will be adequate to determine where health professional learners sit on a spectrum from racist, through to demonstrating culturally safe care. Similarly, we assume that no single model of assessment or educational theory will incorporate this intricacy. As such, we propose that development and assessment of cultural safety can be conceptualised using a model that considers and, to some extent, aligns with: Miller’s pyramid (a commonly used framework for the assessment of clinical skills/performance/competence) [33], the AHPRA definition of cultural safety [19], the continuum of cultural safety [3, 4], Aboriginal ways of knowing, doing, and being [34], transformative learning theory [35], and Gee et al.’s model of social and emotional wellbeing that incorporates historical, social and political determinants of health and wellbeing [26] (Fig. 1).

To explain this model further we start with Miller’s pyramid (the blue triangle in Fig. 1). We use the example of training for an Australian GP fellowship. General practice training in Australia can currently be undertaken through one of five different pathways including the Australian General Practice Training (AGPT) program, Remote Vocational Training Scheme, Rural Generalist, Independent pathway and the General Practice Experience Pathway [36]. Fellowship can be achieved through two colleges, RACGP or ACRRM. RACGP registrars typically train for two to three years in general practice before completing a series of exams, including two written papers – “knows and knows how” and a clinical competency exam – “shows”—where at least one out of  nine stations relate to an Australian Aboriginal and Torres Strait Islander patient with the same marking rubric used for all cases in the exam. RACGP registrars are currently not assessed summatively in the workplace in performance integrated practice (“does”) [37]. In contrast, ACRRM registrars normally complete an additional training year when compared to RACGP registrars [38]. Their assessment includes direct observation of consultations “does” (referred to as mini-CEX) and multi-source feedback (MSF). A registrar is not required to include Australian Aboriginal and Torres Strait Islander patients in this process [38]. Summatively, a Structured Assessment using Multiple Patient Scenarios (best described as a hybrid viva-voce and objective structured clinical examination) is used and will typically involve at least one scenario with the candidate doing outreach clinics to a remote Australian Aboriginal community [38]. These assessments have not been validated with Australian Aboriginal and Torres Strait Islander patients [39, 40]. Recent authors have also proposed an additional layer to Miller's pyramid labelled “is”, reflecting the concept of professional identity formation [41] and in the case of cultural safety, encapsulating ongoing critical reflection. In addition, we propose a further layer at the base of Miller’s pyramid to reflect cultural incapacity or unconscious incompetence [42] as some health professional learners are not aware of their culturally unsafe stance.

Furthermore, using Miller’s pyramid also allows us to demonstrate where terminology regarding cultural safety, or similar, align and ideally minimise confusion (orange triangle in Fig. 1). For example, cultural safety is the “is” and “doing” of Miller’s pyramid whilst cultural competency is the “showing how”. We also invert the pyramid for cultural safety terminology reflecting that we are wanting to place greater value on work-based assessment of cultural safety rather than static knowledge.

Additionally, within the model we overlay a triangle with the Australian AHPRA definition (the yellow triangle in Fig. 1). Within this framework the assessment of cultural safety should be considerate of the complex interplay between health professional learners and the components of the AHPRA definition [19]. The model also illustrates the importance of development and assessment of cultural safety being embedded in Aboriginal ways (the red triangles in Fig. 1). As such, Miller’s pyramid, the AHPRA components of cultural safety, and the continuum of cultural safety are encapsulated within Martin and Mirraboopa’s theoretical framework of Aboriginal ways of knowing (knowledge of history, culture, customs and beliefs), doing (skills and practicing behaviours) and being (critical reflection) [34].

Next, we nominate these components are founded upon a transformative learning ethos where the educational and assessment model aims, as described by Frenk et al. [35], to produce transformative leaders who are “enlightened change agents” (p 1952). Finally, the whole model is dependent on cultural safety being determined by Indigenous peoples and must consider the complexities of health and wellbeing including social, historical, and political determinants of these.

Our model provides a framework to both demonstrate and explore the complexity of cultural safety within a general practice consultation. The AHPRA consensus statement, is very broad, and whilst describing cultural safety, does not provide specific, or measurable attributes to guide registrar assessment. This risks registrar “knowing, doing and being” culturally safe care being ethereal unless further definition of what constitutes culturally safe care is forthcoming. As cultural safety must be determined by Aboriginal and Torres Strait Islander individuals, families and communities, these parameters must be derived and endorsed by the Aboriginal and Torres Strait Islander community. Ensuring Indigenous ownership adds additional complexity to the design and delivery of the assessment of cultural safety when the Aboriginal and Torres Strait Islander community, although viewed as a collectivist society, is a heterogenous society [43] and when it is normative for all patients to impose their individual bias on consultations [28].

In view of this complexity, we will present our research methodology including the development of data collection tools. We will use our proposed model to frame this research. The protocol outlined here will be used in several inter-related studies exploring the research question of “How can cultural safety, as determined by Aboriginal and Torres Strait Islander people, be assessed amongst GP trainees?” We aim to develop a tool to assess whether GP registrars are conducting a culturally safe consultation, where cultural safety is determined by Aboriginal and Torres Strait Islander peoples.

## Methods

### Methodological overview

This study will be situated in a pragmatic philosophical position to explore cultural safety primarily from the Aboriginal and Torres Strait Islander patients’ perspective with triangulation and validation of findings with the GP and GP registrar perspective, the Aboriginal and Torres Strait Islander community, and the medical education community. Epistemologically, pragmatism is based on the premise of focussing on practical understandings of concrete, real-world problems and uses the best available methods to achieve this outcome [44]. This allows for integration of both quantitative and qualitative data to explore complex problems in need of a multi-dimensional approach [45].

### Study design

**Fig. 2:**
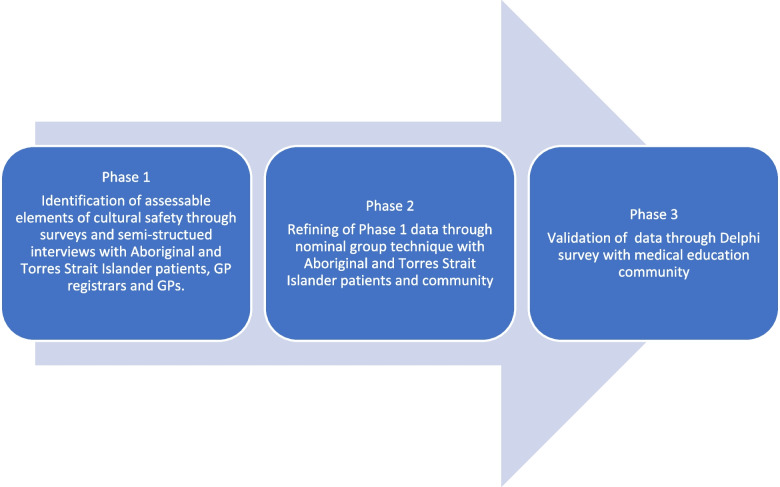
Three phases of the research protocol

This study is funded through an Australian government Medical Research Futures Fund Clinician Researchers grant, and an Australian College of Rural and Remote Medicine educational research grant. A mixed methods sequential embedded design approach [46] will be used to address the research question and sub-questions. This will be in three phases as indicated in Fig. 2.

This approach is chosen to generate data on the complex concept of cultural safety and allow for: (a) identification of culturally safe and unsafe care that can inform cultural safety training; (b) allow for triangulation of patient data with GP registrar and GP data; and (c) refinement and validation of the data with both Aboriginal and Torres Strait Islander peoples and medical education experts.

This study has been built upon the National Health and Medical Research Council guidelines for the ethical conduct of research with Aboriginal and Torres Strait Islander peoples [47]. It was therefore designed with the six values of ethical conduct in research at its core: namely spirit and integrity, responsibility, reciprocity, respect, equity, and cultural continuity [47]. The research was developed in response to a need determined by Aboriginal and Torres Strait Islander health workers and cultural mentors within a regional Aboriginal Medical Service (AMS) (hereafter referred to as the seed AMS) where KB is a practicing GP and RW the chairperson of the organisation. The team has recruited an Indigenous expert panel to ensure these principles and local community protocols are adhered to and has involved health workers, cultural educators, cultural mentors, health advocates and Indigenous academics as part of the research team. Support for the project was sought from, and provided by, the seed AMS staff and board, other Aboriginal Community Controlled Health Organisations, and general practices within the region of study, the National Cultural Educator and Cultural Mentor Network, and the Leaders in Indigenous Medical Education Network (a national group drawn from the university sector).

The James Cook University Human Research Ethics Committee approved this study (H8296) following review by Aboriginal and Torres Strait Ethics Advisors in accordance with the National Health and Medical Research Council guidelines.

### Participants and recruitment

There will be four different groups of participants. Each will be described separately. Participants can withdraw from the study at any time with no consequences. Participants will be remunerated for their time with a gift voucher to the value of $50.

#### Aboriginal and Torres Strait Islander patient participants

Primary care, or GP care, for Aboriginal and Torres Strait Islander patients can be sought through a variety of services including private general practices, and services initiated and operated by Aboriginal and Torres Strait Islander communities for Aboriginal and Torres Strait Islander peoples (Aboriginal Community Controlled Health Organisations or ACCHOs). In this study, self-identifying, adult Aboriginal and Torres Strait Islander patients attending participating private general practices and ACCHOs in Queensland will be invited to participate.

ACCHOs and private general practices, agreeing to participate in the study, are those that have a pre-existing relationship with staff and/or board members at the seed AMS. Senior staff at these practices have been approached to inform them of the study and seek support for the study. Where appropriate, they have been invited to participate in governance structure of the project, through the expert panel. The practices are all within rural and regional Queensland. The three ACCHOs are geographically dispersed being at least 550 kms distant from each other. The private practices are near the seed AMS.

Patients will be invited by practice staff (not in a position of power), either verbally or through providing an information sheet at the end of their consultation, to participate in the study. This may be in the form of a telephone conversation or email if consultation is occurring via telehealth. All patients satisfying inclusion criteria at the ACCHOs will be approached, dependant on practice workflow and demands, until the sample size is achieved. Patients meeting inclusion criteria at the mainstream general practice will be purposively approached until sample size achieved; or a telephone call or letter sent using practice contact details inviting them to participate in the project. Inclusion criteria are: (a) the patient self-identifies as an Aboriginal and Torres Strait Islander person; (b) aged > 18 years; and (c) capacity to give informed consent.

Patients will be asked during the interview if they wish to be invited to participate in Phase 2 of the research project.

#### Registrar participants

One Queensland GP registrar training organisation, James Cook University General Practice Training (JCUGP), agreed to participate in the study after direct approach by the principal investigator, who has existing professional relationships with JCUGP senior staff. Registrars within JCUGP work across Queensland, including the Torres Strait Islands, but excluding the south-east corner of the state [48]. Within JCUGP region approximately 70,000 or two-thirds of Queensland’s Aboriginal and Torres Strait Islander people live in diverse communities – from regional cities to remote islands [48]. Given the diversity of communities, the high population of Aboriginal and Torres Strait Islander peoples, and the number of registrars, sampling only one organisation was considered appropriate. All JCUGP registrars undertake mandatory cultural awareness training modules that are delivered by cultural educators. JCUGP also delivers cultural safety training which is informed by the Aboriginal and Torres Strait Islander Community. Many registrars will gain additional skills and experience throughout their hospital and general practice training.

In this study, all GP registrars undertaking active training with JCUGP will be invited to participate in the study. Registrars could be undertaking training in a range of settings including specialty-based hospital disciplines, rural generalist hospitals, AMSs, and mainstream general practices.

All JCU-GP registrars will be sent an invitation email which will include links to the participant information sheet, consent form and survey. Participants are asked to provide informed consent to completion of both survey and interview. They will be asked to include name and contact details (email or telephone) to allow the research assistant to contact them to schedule the interview. This identifying information will be stored securely and separately to the research data and a unique identifier code used to link the survey and interview data.

#### General practitioner participants

GPs, who have been working at the participating practices (see section *Aboriginal and Torres Strait Islander patient participants*) for more than six weeks, will be invited to participate in the project to explore practices that they perceive are culturally safe. All GPs will be invited by practice staff (not in a position of power), either verbally or through giving of an information sheet (hard-copy or emailed), to participate in the study.

#### Delphi participants

Participants will include Australian Aboriginal and Torres Strait Islander people who are patients, both Indigenous and non-Indigenous patient advocates, experts in Australian Aboriginal and Torres Strait Islander healthcare and health education (including GPs, medical educators, academics, and health workers), and other key stakeholders. Recruitment will occur by a variety of means including word-of-mouth, snowballing and invitation emails through existing networks of the research team and seed AMS.

### Data collection

Data will be collected sequentially in three phases.

#### Phase 1

Phase 1 uses a concurrent embedded mixed methods approach and will gather both survey data and semi-structured interview data to explore the research questions [46]. Data will be collected in three parts, and across three groups of participants (Table 1). In this method, data will be collected and analysed concurrently and results from qualitative data synthesised with quantitative data. To avoid power imbalances and facilitation of frank answers, researchers with no pre-existing relationship to any of the participants, will complete data collection in Phase 1.

**Table 1:** Phase 1 – Data collection methodology

	Aboriginal and Torres Strait Islander patients	GP registrars and GPs
Part 1:	Survey Demographic details	Survey Demographic details Experience Cultural capability measurement tool [49] Measurement of attitude change scale [50] Self-reflection and insight scale [51]
Part 2	Semi-structured interviews exploring patient understanding and experience of cultural safety	Semi-structured interviews exploring registrar understanding of cultural safety
Part 3:	Detailed exploration of patient’s perception of key areas identified in the cultural safety literature	Detailed exploration of registrar’s perception of key areas identified in the cultural safety literature
Number of participants	We are aiming to recruit approximately ten patients from each of the four participating practices, but data collection will continue until the data produces no new insights and data is repeating	We are aiming to recruit approximately twenty GP registrars and three GPs from each of the four participating practices, but data collection will continue until the data produces no new insights and data is repeating

The data collection methodology will be described separately for Aboriginal and Torres Strait Islander patients, GP registrars and GPs. Informed consent will be obtained and recorded in electronic format, on Qualtrics, prior to completing the online survey. The consent will encompass both the survey and the subsequent interview. The research assistant will be available to aid where required.

### Aboriginal and Torres Strait Islander patients

#### Part 1

Part 1 will involve administration of a Qualtrics® based survey considering demographic details of the participating patient, including age and gender. Further questions explore five selected social determinants of health that have been shown to contribute significantly to the health gap between Indigenous and non-Indigenous adults [52]. These determinants are household income, employment and hours worked, highest non-school qualification, level of schooling completed, and housing adequacy [52]. These social determinants are explored recognizing that the interaction between cultural safety, social factors and wellbeing is very complex and a method for assessing cultural safety needs to be cognizant of this relationship. The questions are mirrored from the Australian Bureau of Statistics census data [53].

#### Part 2

Part 2 will involve semi-structured interviews with patients to explore their understanding of cultural safety. The semi-structured interview guide was developed *de-novo* being informed by the literature, experience, and advice from a community advisory panel of Aboriginal and Torres Strait Islander people who are overseeing the project (Additional file 1: Appendix 1). One question asks patients to choose their preferred GP from 45 photographic different face images. This is designed to explore potential patient bias in a consultation. These images represent a diversity of gender, age, ethnicity, and appearance. Images include Aboriginal and Torres Strait Islander doctors, obtained from Indigenous health websites, and others from a free face generator (thispersondoesnotexist.xyz) [54].

The interview guide was reviewed by the whole research team, two independent GPs, and members of the community advisory panel. The survey and interview guide were piloted with two Aboriginal health workers. Interviews are expected to take 30–45 min. Participants will be offered face-to face or remote interviews, either video or audio only. Interviews will be recorded following confirmation of informed consent and then electronically transcribed. These transcripts will be checked for accuracy by a research assistant.

#### Part 3

At the end of semi-structured interviews, the interviewer will request patients rate the importance, from not important (one) to very important (five), of several factors when consulting their GP. These factors where identified from Australian medical education literature and included sociocultural differences [55], the importance of general consultation and communication skills, the ability to listen, respect, trust, and self-reflection [6]. Patients will be encouraged to justify or explain their response regarding the importance of knowing Australian history pre-colonisation, the experiences of Aboriginal and Torres Strait Islander people after colonisation and having medical knowledge and skills. In addition, they are asked to rate the importance of eye contact, the value of silence, the use of some traditional language, inclusion of spirituality in a consultation and the importance of including family, elders of other significant others in the consultation. Finally, participating patients will be asked to consider how important their own culture is to them and their identity and the importance of their own connection to land.

### GP registrars and GPs

#### Part 1

Like patients, part 1 will involve administration of a Qualtrics®-based survey considering demographic details of the GP registrars including age, gender, post-graduate level, stage of training, training college, university of graduation, time lived in Australia, exposure to Aboriginal and Torres Strait Islander patients and type of current practice (AMS, hospital, or mainstream general practice). This information will be used to describe the participants, to determine if participants are representative of the JCUGP registrar cohort, and to provide context for qualitative data analysis.

The survey will include questions exploring the “being” and “is” of cultural safety regarding attitude and beliefs for comparison with the qualitative data. Numerous self-assessment tools exist to measure a learner’s behaviour and attitude [56]. Validated survey questions from West et al.’s cultural capability measurement tool (developed for nurses) [56] and Ryder et al.’s measurement of attitude change [50] has been utilised. Ryder et al. developed and validated a questionnaire to measure attitude change in health professionals (including medical students) following completion of a cultural safety training program [48]. Both questionnaires by West et al. [56] and Ryder et al. [50] occur outside of the context of patient interaction and are self-assessed measures of attitude. The questions asked in both surveys share similarities and therefore, the questions were compared and rationalised by choosing one representative question from overlapping queries (Additional file 1: Appendix 2). As Ryder et al.’s research included medical students these questions were preferentially used. Wording of the survey is modified such that ‘health professional’ or ‘student’ is replaced with ‘GP, ‘Aboriginal people’ is broadened to ‘Aboriginal and Torres Strait Islander people’, ‘patient’ is used instead of ‘client’, “GP consultation” substituted for “hospitalised patient’s room” and “Prohibited” replaced with “limited or restricted” to avoid an ‘all or nothing’ type of question.

We altered the West et al. survey question: “Aboriginal and Torres Strait Islander peoples receive special treatment from government” to “Aboriginal and Torres Strait Islander peoples receive *unnecessary* special treatment from government”. Investigators felt the original question could be interpreted by participants in this study as a knowledge assessment—about Australian government initiatives to close the gap on Indigenous health disparities [57] rather than a reflection of participants’ attitude.

To consider registrar self-reflection, we examined 21 different questionnaires identified in a systematic search by Soemantri et al. [51]. Five questions from the Self-Reflection and Insight Scale (SRIS), looking at intention for reflection, were chosen by the research team as most appropriate and incorporated [58] into the final survey (Additional file 1: Appendix 2).

#### Part 2

Like patients, Part 2 will involve semi-structured interviews with GP registrars and GPs to explore their understanding of cultural safety. The semi-structured interview guide was developed in the same manner as the patient guide and is presented in Additional file 1: Appendix 3.

The survey and interview guide were piloted with two GP registrars and a recent GP fellow. Interviews are expected to take 30–45 min.

The interview will be conducted at least 5 days after the survey to minimise survey questions influencing responses in the semi-structured interviews. Participants will be offered face-to face or remote interviews, either video or audio only. Interviews will be recorded following confirmation of informed consent and then electronically transcribed. These transcripts will be checked for accuracy by a research assistant.

#### Part 3

Part 3, at the end of semi-structured interviews, the interviewer will request GP registrars and GPs to rate the same questions presented to the patient participants.

#### Phase 2

Findings from Phase 1 will be validated using a two-step qualitative approach to confirm essential elements of any potential cultural safety assessments in GP consultations.

Phase 2 will utilise an adapted nominal group technique (NGT). McMillan et al. [59] detailed a simplified model of NGT (silent generation, round robin, clarification, ranking and discussion). This project will incorporate the Indigenous research approach of yarning [60] in place of discussion. Bessarab and Ng’andu [60] describe research topic yarning as an “*informal and relaxed discussion through which both the researcher and participant journey together visiting places and topics of interest relevant to the research study*”. It will also adopt similar methods as described by Woolley [61] to encourage discussion and debate on inclusion of elements from Stage 1 in a potential assessment tool:

*“The Yarning Circle discussion involved the facilitator asking the other participants to describe any specific skills, knowledge or attitudes they felt were important…Participant comments under each…heading were captured as a phrase or statement on butcher’s paper in front of the group so that participants were able to see the ideas generated. Visual representation of the data generated in the focus group enabled participants to come to an agreement about how each comment was summarised”.*

As a primary aim for the project will be to develop a patient-driven assessment tool, only Australian Aboriginal peoples will be invited to consider element inclusion/exclusion in the first instance. Australian Aboriginal patients participating in Stage 1 will be invited to participate in the adapted NGT group. Other participants will be purposively sampled, though the networks of both the research team and participating practices and invited to participate. These will include additional Australian Aboriginal patients and Australian Aboriginal representatives from key stakeholder groups, including community. Once participants are known, advice will be sought from the expert panel regarding appropriateness of separate groups for patients and stakeholders to minimise any potential power differential. Snowball recruiting will be encouraged.

Ideally the adapted NGT will be conducted face-to-face but could be managed through an online meetings platform if circumstances require this approach. Following a written and verbal informed consent process, adapted NGT groups will be audio-recorded and transcribed as per individual semi-structured interviews and any written material collated or photographed.

### Sample size

A maximum group size of seven has been recommended for NGT [59]. One to five adapted NGT groups will be conducted in geographically diverse locations. The number of adapted NGT will be dependent on number and scheduling requirements of participants.

#### Phase 3

Phase 3 involves a Delphi survey to further validate the findings of Phase 1 and 2. A Delphi technique (DT) uses a multi-stage process of anonymous questionnaires to create a highly structured group interaction [59]. Elements identified for inclusion in an assessment approach from Phase 2 adapted NGT will be collated and refined into a Delphi questionnaire. Links to consent and DT questionnaires will be emailed to participants and reminders sent to all participants. Qualtrics® will be used to administer the questionnaire online and collate responses electronically. Elements will be rated by participants on a Likert scale and free-text comments written to justify their response. The process will be repeated until consensus is reached about the elements which are important to include in an assessment of cultural safety.

### Sample size

A panel size of 15 is suggested as optimal size for this technique [59].

### Data analysis

Survey data will be descriptively analysed using Excel® to both characterise the participants and provide contextual data for assisting in interpreting the interview data. One researcher (KB) will analyse all interview and adapted NGT transcripts through a content analysis approach using theory-driven codes derived from the AHPRA definition of cultural safety (free from racism, knowledge, skills, attitude, behaviours, power differential) and emerging data-driven codes. NVivo® analysis software will be used when coding data, recording frequency of occurrence of item of interest, and collating key concepts. Interview data and coding will be checked and reviewed by other researchers and the research team will meet frequently to reflect and debrief to support the dependability and credibility of the data analysis.

This project will maintain transparency through the research process by input from the community panel, regular discussion with the seed AMS staff, peer examination of the data through conference presentations, ongoing journaling of personal reflexivity on the data, and identifying disconfirming evidence that is contrary to evidence supporting a theme. Multiple reviews of coding will be conducted to ensure agreement in the coding and to minimise bias of any individual researcher. In addition, KB will work with the community advisory panel during phases of thematic analysis and coding, further minimising the potential bias associated with the individual researcher.

Consensus elements from the three phases will be synthesised into a potential assessment model. Further research beyond this project will be required to pilot and validate the proposed assessment approach.

### Reflexivity

The principal investigator, KB, is an experienced GP academic working in the seed AMS in south-east Queensland. Her cultural heritage is uncertain and is impacted by the complexities surrounding Aboriginal identity within Australia [62]. RW is an Aboriginal academic from Kunja Nations, NH an Aboriginal and Torres Strait Islander cultural educator for JCUGP, RE a senior researcher, TS and HW academic GPs, and LM an academic GP. A community advisory group of Aboriginal and Torres Strait Islander people, associated with the seed AMS, have been involved in the research since inception.

## Discussion

This study will be one of the first to explore how cultural safety, as determined by Aboriginal and Torres Strait Islander peoples, can be assessed in general practice consultations. The study will explore how GPs and GP registrars perceive cultural safety with Aboriginal and Torres Strait Islander patients and alignment with the community derived AHPRA definition of cultural safety. As such we will compare the GP and GP registrar data and patient data to identify the concordance with each other and the AHPRA definition to help shape teaching and assessment of cultural safety.

This protocol is shared to stimulate awareness and discussion around this significant issue and prompt other studies in this area. We hypothesise that conceptualising the assessment of cultural safety through the multiple dimensions of a community-derived definition [19], the continuum of cultural safety [3, 4], educationally (using Miller’s pyramid [33] and transformative learning theory [35]), Aboriginal ways of knowing, doing, and being [34], and social and emotional wellbeing [26] will allow an assessment outcome to reflect the complexity of cultural safety within general practice.

Key limitations to this study include that we are studying a relatively small number of participants in a geographically discrete region. While there are strengths in this geographically discrete approach, especially regarding specificity to the local cultural context, outcomes of this project should be assessed for wider application. In addition, we are relying on participants to self-describe their behaviours, and attitudes. With the GP and GP registrar surveys, responses in these questionnaires may not accurately reflect behaviour, or the opinion and experience of the patient [63]. Direct observation of practice may provide further insight into attributes of cultural safety.

This project has implications for practice and training of medical professionals both within Australia and internationally. Within Australia, culturally safe practice has been recognised by both the RACGP and ACRRM as a priority for inclusion in training programs. This protocol allows the exploration of cultural safety, as understood by patients, GPs, and registrars, and to identify gaps between the knowing, doing and being of general practice. This understanding is vital to allow the shaping and improvement of cultural safety training within GP training curricula and consideration of assessment of GP registrars knowing, showing, and doing.

However, this protocol also has potential within a wider context. Other areas of healthcare, including other medical specialities, allied health, dental and nursing, could benefit from similar studies exploring cultural safety amongst trainees. In the same way, an exploration of the process of developing safe care for culturally diverse peoples has potential benefits internationally. It is hoped that the sharing of this protocol offers opportunities to expand the knowledge base around culturally safe care more widely.

## Supplementary Information

**Additional file 1:** **Appendix 1.** Semi-structured interview guide for patients. **Appendix 2.** Survey questions for GPs and GP registrars. **Appendix 3.** Semi-structured interview guide for GPs and GP registrars.

## Data Availability

The datasets generated and/or analysed during the current study are not publicly available due to reasons of sensitivity but are available from the corresponding author on reasonable request.

## References

[1] Truong M, Paradies Y, Priest N. Interventions to improve cultural competency in healthcare: a systematic review of reviews. BMC Health Serv Res. 2014;14:99. 10.1186/1472-6963-14-99 PMC3946184 24589335

[2] MacKean T, Fisher M, Friel S, Baum F (2019). A framwork to assess cultural safety in Australian public policy. Health Promot Int.

[3] Ramsden I (2002). Cultural safety and nursing education in Aotearoa and Te Waipounamu.

[4] Mashford- Pringle A, Skura C, Stutz S, Yohathasan T. What we heard: Indigenous Peoples and COVID-19. Waakebiness-Bryce Institute for Indigenous Health. Dalla Lana School of Public Health: University of Toronta; 2021.

[5] Paul D, Hill S, Ewen S. Revealing the (in)competency of "cultural competency" in medical education. AlterNative. 2012;8(3):318–28.

[6] Brumpton K, Evans R, Ward R, Sen Gupta T. A consistent definition of cultural safety within Australian health professional education: a scoping review. AlterNative. 2022:18(3):436–44.

[7] Grant J, Parry Y, Guerin P (2013). An investigation of culturally competent terminology in healthcare policy finds ambiguity and lack of definition. Aust N Z J Public Health.

[8] McKivett A, Paul D, Hudson N (2019). Healing conversations: developing a practical framework for clinical communication between Aboriginal communities and healthcare practitioners. J Immigr Minor Health.

[9] Liaw ST, Hasan I, Wade V, Canalese R, Kelaher M, Lau P (2015). Improving cultural respect to improve Aboriginal health in general practice: A multi-methods and multi-perspective pragmatic study. Aust Fam Physician.

[10] Freeman T, Edwards T, Baum F, Lawless A, Jolley G, Javanparast S (2014). Cultural respect strategies in Australian Aboriginal primary health care services: Beyond education and training of practitioners. Aust N Z J Public Health.

[11] Barnett L, Kendall E (2011). Culturally appropriate methods for enhancing the participation of Aboriginal Australians in health-promoting programs. Health Promot J Austr.

[12] Smith K, Fatima Y, Knight S (2017). Are primary healthcare services culturally appropriate for Aboriginal people? Findings from a remote community. Aust J Prim Health.

[13] Bennett B, Redfern H, Zubrzycki J (2018). Cultural responsiveness in action: Co-constructing social work curriculum resources with Aboriginal communities. Br J Soc Work.

[14] Chang ES, Simon M, Dong X (2012). Integrating cultural humility into health care professional education and training. Adv Health Sci Educ.

[15] Chapman R, Martin C, Smith T (2014). Evaluation of staff cultural awareness before and after attending cultural awareness training in an Australian emergency department. Int Emerg Nurs.

[16] Chong A, Renhard R, Wilson G, Willis J, Clarke A (2011). Improving Cultural Sensitivity to Indigenous People in Australian Hospitals a Continuous Quality Improvement Approach. Focus on Health Professional Education : A Multi-disciplinary Journal.

[17] Isaacs AN, Raymond A, Jacob E, Jones J, McGrail M, Drysdale M (2016). Cultural desire need not improve with cultural knowledge: A cross-sectional study of student nurses. Nurse Educ Pract.

[18] Omeri A, Ahern M (1999). Utilising culturally congruent strategies to enhance recruitment and retention of Australian indigenous nursing students. J Transcult Nurs.

[19] Ahpra & National Boards (2019). A consistent baseline definition of cultural safety for the National Scheme.

[20] Australian Bureau of Statistics (2021). Aboriginal and Torres Strait Islander people: census - reference period 2021. https://www.abs.gov.au/statistics/people/aboriginal-and-torres-strait-islander-peoples/aboriginal-and-torres-strait-islander-people-census/2021. ABS: Canberra; 2022. Accessed 30 Apr 2023.

[21] Australian Government Department of Health and Aged Care. Status and determinants of Aboriginal and Torres Strait Islander health. https://www.health.gov.au/topics/aboriginal-and-torres-strait-islander-health/status-and-determinants. DOHA: Canberra; 2021. Accessed 30 Apr 2023.

[22] Ahpra & National Boards (2020). The National Scheme’s Aboriginal and Torres Strait Islander health and cultural safety strategy 2020–2025.

[23] Australian Institute of Health and Welfare (2022). Cultural safety in health care for Indigenous Australians: monitoring framework.

[24] Australian Medical Council Limited. Review of the accreditation standards for primary medical programs. https://www.amc.org.au/review-of-accreditation-standards-for-primary-medical-programs-medical-schools/. AMC; 2022. Accessed 30 Apr 2023.

[25] Anderson IP, Robson BP, Connolly MMPH, Al-Yaman FP, Bjertness EP, King AMD (2016). Indigenous and tribal peoples' health (**The Lancet**-Lowitja Institute Global Collaboration): a population study. Lancet.

[26] Gee G, Dudgeon P, Schultz C, Hart A, Kelly K. Social and emotional wellbeing and mental health: an Aboriginal perspective. Chapter 4, In: Dudgeon P, Milroy M, Walker R,( editors). Working together: Aboriginal and Torres Strait Islander Mental Health and Wellbeing Principles and Practice – Revised Ed., in National strategic framework for Aboriginal and Torres Strait Islander Peoples’ mental health and social and emotional wellbeing 2017–2023. Revised ed. https://www.niaa.gov.au/sites/default/files/publications/mhsewb-framework_0.pdf. Canberra: Department of Prime Minister and Cabinet; 2017. p. 55. Accessed 30 Apr 2023.

[27] Royal Australian College of General Practitioners (2020). Standards for general practices.

[28] Brumpton K, Sen Gupta T, Evans R, Ward R (2022). Assessment of cultural safety in a post-Objective Structured Clinical Examination (OSCE) era. AJGP.

[29] Royal Australian College of General Practitioners (2022). 2022 RACGP curriculum and syllabus for Australian general practice.

[30] Australian College of Rural and Remote Medicine (2020). Rural generalist curriculum.

[31] Australian Medical Council Limited. Proposed graduate outcome statements: draft for consultation. https://www.amc.org.au/wp-content/uploads/2022/09/ATTACHMENT-A-Proposed-Graduate-Outcome-Statements-Draft-for-consultation-August-2022.pdf. Canberra: AMC; 2022. Accessed 30 Apr 2023.

[32] Watt K, Abbott P, Reath J (2016). Developing cultural competence in general practitioners: an integrative review of the literature. BMC Fam Pract.

[33] Cruess R, Cruess S, Steinert Y (2016). Amending Miller's pyramid to include professional identity formation. Acad Med.

[34] Martin K, Mirraboopa B (2003). Ways of knowing, being and doing: a theoretical framework and methods for Indigenous and Indigenist re-search. J Aust Stud.

[35] Frenk J, Chen L, Bhutta ZA, Cohen J, Crisp N, Evans T (2010). Health professionals for a new century: transforming education to strengthen health systems in an interdependent world. Lancet.

[36] General Practice Registrars Australia. Pathways into general practice. https://gpra.org.au/training/fellowship-pathways/ GPRA; 2022. Accessed 30 Apr 2023.

[37] Royal Australian College of General Practitioners. Examination guide. https://www.racgp.org.au/educa tion/registrars/fracgp-exams/exam-support-program-resources/examination-guide. Melbourne: RACGP; 2022. Accessed 30 Apr 2023.

[38] Australian College of Rural and Remote Medicine A. Fellowship assessment handbook. https://www.acrrm.org.au/docs/default-source/all-files/handbook-fellowship-assessment.pdf?sfvrsn=42ba86eb_8. Brisbane: ACRRM; 2019. Accessed 30 Apr 2023.

[39] Sen Gupta T, Campbell D, Chater A, Rosenthal D, Saul L, Connaughton K, et al. Fellowship of the Australian College of Rural & Remote Medicine (FACRRM) assessment: a review of the first 12 years. MedEdPublish. 2020;9(100). 10.15694/mep.2020.000100.1 PMC10712634 38090052

[40] Brouwers M, Custers J, Bazelmans E, Van Weel C, Laan R, van Weel-Baumgarten E. Assessment of medical students’ integrated clinical communication skills: development of a tailor-made assessment tool. BMC Med Educ. 2019;19(118):1–10. 10.1186/s12909-019-1557-3 PMC6489308 31035995

[41] Al-Eraky M, Marei H (2016). A fresh look at Miller's pyramid: assessment at the 'Is' and 'Do" levels. Med Educ.

[42] Broadwell M (1969). Teaching for learning (XVI). The Gospel Guardian.

[43] Jalla C, Hayden G. Aboriginal health research is not black and white - lessons from the field. Australian Indigenous HelathBulletin. 2014;14(3).

[44] Patton M (2005). Qualitative Research & Evaluation Methods.

[45] Allemang B, Sitter K, Dimitropoulos G (2022). Pragmatism as a paradigm for patient-oriented research. Health Expectat.

[46] Creswell J, Plano CV (2007). Designing and conducting mixed methods research.

[47] National Health and Medical Research Council (2018). Ethical conduct in research with Aboriginal and Torres Strait Islander Peoples and communities: guidelines for researchers and stakeholders.

[48] James Cook University. General Practice training. Townsville: JCU; 2022. https://www.jcugp.edu.au/. JCU: Townsville; 2022. Accessed April 30, 2023.

[49] West R, Wrigley S, Mills K, Taylor K, Rowland D, Creedy D (2017). Development of a First Peoples-led cultural capability measurement tool: a pilot study with midwifery students. Women Birth.

[50] Ryder C, MacKean T, Ullah S, Burton H, Halls H, McDermott D (2019). Development and validation of a questionnaire to measure attitude change in health professionals after completion of an Aboriginal health and cultural safety training programme. Aust J Indigenous Educ.

[51] Soemantri D, Mccoll G, Dodds, A. Measuring medical students’ reflection on their learning: modification and validation of the motivated strategies for learning questionnaire (MSLQ). BMC Med Educ. 2018;18(1):1–10. 10.1186/s12909-018-1384-y PMC6251170 30466427

[52] Australian Institute of Health and Welfare. Determinants of health for Indigenous Australians. https://www.aihw.gov.au/reports/australias-health/social-determinants-and-indigenous-health health. Canberra: AIHW; 2020.

[53] Australian Bureau of Statistics (2016). Census household form.

[54] Vincent J. ThisPersonDoesNotExist.com uses AI to generate endless fake faces: Vox Media; 2019. Available from: https://www.theverge.com/tldr/2019/2/15/18226005/ai-generated-fake-people-portraits-thispersondoesnotexist-stylegan. Accessed 30 Apr 2023.

[55] Jongen C, McCalman J, Bainbridge R, Clifford A (2018). Cultural Competence in Health - A review of the Evidence.

[56] West R, Mills K, Rowland D, Creedy DK (2018). Validation of the first peoples cultural capability measurement tool with undergraduate health students: a descriptive cohort study. Nurse Educ Today.

[57] Productivity Commission. Closing the gap annual data compilation report July 2022. https://www.pc.gov.au/closing-the-gap-data/annual-data-report/report/closing-the-gap-annual-data-compilation-report-july2022.pdf. Canberra: Commonwealth of Australia, Office of the Prime Minister and Cabinet; 2022. Accessed 30 Apr 2023.

[58] Naeimi L, Abbaszadeh M, Mirzazadeh A, Sima A, Nedjat S, SH (2019). Validating self-reflection and insight scale to measure readiness for self-regulated learning. J Educ Health Promotion.

[59] McMillan S, King M, Tully M (2016). How to use the nominal group and Delphi techniques. Int J Clin Pharm.

[60] Bessarab D, Ng'andu B (2010). Yarning about yarning as a legitimate method in Indigenous research. Int J Crit Indig.

[61] Woolley T, Sivamalai S, Ross S, Duffy G, Miller A (2013). Indigenous perspectives on the desired attributes of medical graduates practising in remote communities: a Northwest Queensland pilot study. Aust J Rural Health.

[62] Carlson B (2016). The politics of identity: who counts as Aboriginal today?.

[63] Carr S, Johnson, P. Does self reflection and insight correlate with academic performance in medical students? BMC Med Ed. 2013;13(113):113–7. 10.1186/1472-6920-13-113 PMC3765283 23971859

