# Supplementary material for: Assessing cultural safety in general practice consultations for Indigenous patients: protocol for a mixed methods sequential embedded design study
Source: BMC Med Educ. 2023 May 2;23:306. doi: 10.1186/s12909-023-04249-6 (PMC10152729; doi:10.1186/s12909-023-04249-6)
Supplement: Supplementary file 1 — Additional file 1: Appendix 1. Semi-structured interview guide for patients. Appendix 2. Survey questions for GPs and GP registrars. Appendix 3. Semi-structured interview guide for GPs and GP registrars. [file 12909_2023_4249_MOESM1_ESM.docx]

# Appendices

Appendix 1 - Semi-structured interview guide for patients

| **Research question** | **Inquiry purpose** | **Main interview question** | **Probing questions** |
| --- | --- | --- | --- |
| How do patients perceive cultural safety in a general practice consultation? | Determine how patients view cultural safety for comparison with the AHPRA definition and GP data. | We would like to know, in your opinion, what makes a good doctor? | Can you think a GP that you like? Why do you like them? |
|  |  | How is culture important to you, particularly, when seeking healthcare from a GP? |  |
| Which of the components of the AHPRA definition of cultural safety are identified by patients as important in a GP consultation? | Attitude | Still thinking about a good doctor, in your opinion, how would you describe their attitude? | For example, are they patient, positive, funny? |
|  |  | I am interested in learning about what it means to be respectful. What should a doctor do to show they respect you? | Can you tell me about some examples of ways in which you have been treated with respect by your doctor?  Can you tell me about some examples of ways in which you have been treated disrespectfully by your doctor? |
|  | Practicing behaviours | When seeing your GP, what are the things you like them to do to make you feel like you are being treated well? |  |
|  |  | As an Aboriginal and Torres Strait Islander person, what makes you feel safe in a consultation? |  |
|  |  | When a doctor asks you where you are from, where is your country or your mob, what do you think? | How does this make you feel? |
|  | Power differentials and racism | Unfortunately, sometimes people have bad experiences with a doctor. Can you think of a time when you saw a GP, and this happened to you? | What was this doctor like? How would you describe their attitude? |
|  |  | Sadly, we also know racism and discrimination can occur during GP consultations. Have you ever had an experience with a GP when you felt they were being racist or discriminating against you or your family? | Sometimes patients will describe feeling controlled or punished by their GP. Have you had experiences like this? |
|  |  | Here is a page of photos of peoples’ faces. Let’s imagine all these people are GPs working here today and you must make an appointment for you or a family member. All these doctors have the same level of skill and experience. Can you please choose which doctor you would prefer to see? | Why did you choose that doctor?  Are there any doctors that you will not see? Why? |

Appendix 2 - Survey questions for GPs and GP registrars

| Origin | Item | Item definition |
| --- | --- | --- |
| Ryder et al (51) | CSTQ1 | I think my beliefs and attitudes are influenced by my culture. |
|  | CSTQ2 | A GPs’ own cultural beliefs influence their health care decisions. |
|  | CSTQ3 | Time in the GP curriculum devoted to the promotion of GP self-awareness and well-being is time well spent. |
|  | CSTQ4 | A GPs’ ability to communicate with patients is as important as their ability to solve clinical problems. |
|  | CSTQ5 | The presence of more than two family members in a GP consultation is disruptive to staff and should be limited or restricted. |
|  | CSTQ6 | The quality of patient care could possibly be compromised if a GP is oblivious to the family’s cultural attributes and values. |
|  | CSTQ7 | As a GP if I needed more information about a person’s culture to provide a service, I would feel comfortable asking the person or one of their family members. |
|  | CSTQ8 | Aboriginal and Torres Strait Islander people, due to the own cultural beliefs and values, have the poorest health status in Australia. |
|  | CSTQ9 | Aboriginal and Torres Strait Islander people, should take more individual responsibility for improving their own health. |
|  | CSTQ10 | The Western medical model is sufficient in meeting the health needs of all people including Aboriginal and Torres Strait Islander peoples. |
|  | CSTQ11 | All Australians need to understand Aboriginal and Torres Strait Islander history and culture. |
|  | CSTQ12 | Aboriginal and Torres Strait Islander people should not have to change their culture just to fit in. |
|  | CSTQ13 | We practice equity in the provision of healthcare by treating Aboriginal and Torres Strait Islander people the same as all other patients. |
|  | CSTQ14 | I need to think beyond the individual when considering Aboriginal and Torres Strait Islander health issues. |
|  | CSTQ15 | I have a social responsibility to work for changes in Aboriginal and Torres Strait Islander health. |
| West et al. (57) | W1 | History does not impact on Aboriginal and Torres Strait Islander health. |
|  | W2 | Understanding Aboriginal and Torres Strait Islander peoples’ history will inform my practice as a GP. |
|  | W3 | Understanding Aboriginal and Torres Strait Islander peoples’ social practices will not apply to my practice. |
|  | W4 | I find it difficult to understand the beliefs of different cultural groups. |
|  | W5 | Evidence from research can help me in my practice in Aboriginal and Torres Strait Islander peoples’ health. |
|  | W6 | Aboriginal and Torres Strait Islander peoples receive unnecessary special treatment from government. |
| Soemantri et al (52) | SR1 | I do not often think about my thoughts. |
|  | SR2 | I am not really interested in analyzing my behavior. |
|  | SR3 | It is important for me to evaluate the things that I do. |
|  | SR4 | I am very interested in examining what I think about. |
|  | SR5 | I do not really think about why I behave in the way that I do. |

Appendix 3 - Semi-structured interview guide for GPs and GP registrars

| **Research question** | **Inquiry purpose** | **Main interview question** | **Probing questions** |
| --- | --- | --- | --- |
| How do GP registrars define cultural safety? | Determine how registrars define cultural safety for comparison with the AHPRA definition. | Could you please define for me the concept of cultural safety? | What does a culturally safe consultation look like to you? |
| How do GP registrars develop cultural safety? | To explore how registrars perceive they have developed cultural safety | How do you feel you have developed cultural safety? |  |
|  |  | What has been the most effective way for you to develop this cultural safety? | What cultural safety/competency (or similar) training have you completed?  What are the main things you learnt from this training? |
| What do registrars view as unique to their consultations with Aboriginal and Torres Strait Islander people? | To allow registrars to express the perceived positives of their interaction with Aboriginal and Torres Strait Islander patients. | Why do you think Aboriginal and Torres Strait Islander patients choose (would choose) * to see you as their doctor? |  |
|  | To explore if registrars modify their consultation approach with Aboriginal and Torres Strait Islander patients. | How do you (would you) * describe your consultation approach to Aboriginal and Torres Strait Islander patients? | Do you (would you) use a particular model of consultations?  Do you modify this model in any way for Aboriginal and Torres Strait Islander patients?  Is this different to your approach with patients who do not identify as Aboriginal and Torres Strait Islander? |
| Which of the components of the AHPRA definition of cultural safety are identifiable by a GP registrar? | To individually explore elements of the AHPRA definition and how the registrar and how the registrar perceives they deliver culturally safe care |  |  |
|  | Knowledge | Could you please tell what you know about early Australian history? |  |
|  | Skills | What are the most important things you do (could do)* to make Aboriginal and Torres Strait Islander patients feel culturally safe when you are consulting? |  |
|  | Attitude | How do you demonstrate respect for your Aboriginal and Torres Strait Islander patients? |  |
|  | Practicing behaviours | What is (would be)* your communication strategy in consultations with Aboriginal and Torres Strait Islander patients? |  |
|  |  | How do you demonstrate emotional support for your Aboriginal and Torres Strait Islander patients? |  |
|  | Power differentials and racism | Can you describe a time when you feel a patient may have felt culturally unsafe when you were consulting? |  |
|  | Free of racism | Can you describe Aboriginal and Torres Strait Islander patients for me please? | Tell me about your Aboriginal and Torres Strait Islander patients. What are they like? Do you feel they are different to your non-Indigenous patients? |
|  |  | Can you describe any occasions when you felt controlled or uncomfortable with your Aboriginal and Torres Strait Islander patients? |  |
